# Machine Learning in Predicting Cardiac Events for ESRD Patients: A Framework for Clinical Decision Support

**DOI:** 10.3390/diagnostics15091063

**Published:** 2025-04-22

**Authors:** Chien-Wei Chuang, Chung-Kuan Wu, Chao-Hsin Wu, Ben-Chang Shia, Mingchih Chen

**Affiliations:** 1 Graduate Institute of Business Administration, Fu Jen Catholic University, New Taipei City 242062, Taiwan; 410088032@m365.fju.edu.tw (C.-W.C.); wujs@kttc.com.tw (C.-H.W.); 025674@mail.fju.edu.tw (B.-C.S.); 2 Artificial Intelligence Development Center, Fu Jen Catholic University, New Taipei City 242062, Taiwan; 3 Division of Nephrology, Shin Kong Wu Ho-Su Memorial Hospital, Taipei 111045, Taiwan; 091772@mail.fju.edu.tw; 4 Dialysis Access Management Center, Shin Kong Wu Ho-Su Memorial Hospital, Taipei 111045, Taiwan; 5 School of Medicine, Fu Jen Catholic University, New Taipei City 242062, Taiwan

**Keywords:** machine learning, artificial intelligence, medical decision-making, ESRD, risk factor

## Abstract

**Background/Objectives:** Patients with end-stage renal disease (ESRD) are at an increased risk of major adverse cardiac events (MACEs), highlighting the need for accurate risk prediction and personalized interventions. This study aims to develop and evaluate machine learning (ML) models to identify key predictive features and enhance clinical decision-making in MACE risk assessment. **Methods:** A dataset comprising 84 variables, including patient demographics, laboratory findings, and comorbidities, was analyzed using CatBoost, XGBoost, and LightGBM. Feature selection, cross-validation, and SHAP (SHapley Additive exPlanations) analyses were employed to improve model interpretability and clinical relevance. **Results:** CatBoost exhibited the highest predictive performance among the models tested, achieving an AUC of 0.745 (0.605–0.83) with balanced sensitivity and specificity. Key predictors of MACEs included antiplatelet use, the grade of left ventricular hypertrophy, and serum albumin levels. SHAP analysis enhanced the interpretability of model outputs, supporting clinician-led risk stratification. **Conclusions:** This study highlights the potential of ML-based predictive modeling to improve MACE risk assessment in patients with ESRD. The findings support the adoption of ML models in clinical workflows by integrating explainable AI methods to enable individualized treatment planning. Future integration with electronic health record systems may facilitate real-time decision-making and enhance patient outcomes.

## 1. Introduction

The burgeoning field of machine learning offers promising avenues for enhancing patient care, particularly for those suffering from ESRD, who are at a high risk of MACEs and consequently experience elevated postoperative mortality rates. This study utilizes a range of advanced machine learning algorithms to systematically evaluate predictive features for MACEs in ESRD patients to improve clinical decision-making and patient outcomes. By assessing multiple models, including gradient boosting and deep learning techniques, we aim to address several critical research questions: identifying the most significant predictive features for MACEs, comparing the efficacy of machine learning models with traditional statistical approaches, and analyzing the clinical implications of these findings. This study fills a critical gap in the existing literature by comprehensively evaluating various machine learning models. It aims to contribute actionable insights that could lead to more personalized care and effective management strategies for this vulnerable patient group. In light of these advancements, this study aims to expand the feature set beyond traditional variables and leverage a broader set of contemporary machine learning algorithms to deepen the understanding of risk factors for MACEs in ESRD patients.

Researchers have previously explored the application of machine learning techniques in ESRD patients. For example, Mezzatesta et al. (2019) employed traditional machine learning models such as Logistic Regression (LGR), K-Nearest Neighbors (KNN), Classification and Regression Trees (CARTs), Naive Bayes (NB), and Support Vector Machines (SVCs) to predict cardiovascular diseases in dialysis patients, demonstrating the potential of machine learning methods in predicting MACEs [1]. However, their study was limited in variables, utilizing only 24 features for model construction. With the rapid development of medical and data analysis technologies in recent years, more clinical variables have been identified as closely related to MACEs, presenting new opportunities and challenges for accurately predicting risks in ESRD patients.

To more comprehensively explore risk factors for ESRD patients, this study has expanded the scope of variable collection and employs a variety of novel machine learning algorithms, including Logistic Regression (LGR), Classification and Regression Trees (CARTs) [2], Random Forests (RFs) [3], Extreme Gradient Boosting (XGBoost) [4], CatBoost [5,6], and LightGBM [7]. These methods, which have been proven in recent years to have stronger predictive performance and generalization capabilities, allow for a systematic evaluation that seeks to uncover additional potential predictive factors. By improving the precision of MACE risk assessments, our approach aims to support more effective, personalized care plans for ESRD patients, thereby advancing the field of clinical risk assessment.

In recent years, significant advancements have been made in predicting and identifying risk factors for MACEs in ESRD patients. This article reviews key findings in the field, highlighting the potential and challenges of applying machine learning in this area. Sharma et al. (2013) identified impaired thrombolytic function as a novel risk factor in ESRD, strongly associated with cardiovascular events, underscoring the importance of evaluating thrombus formation and dissolution mechanisms in these patients [8]. Similarly, AlJaroudi et al. (2019) demonstrated that a blunted pre-transplant heart rate response in asymptomatic ESRD patients could predict post-transplant MACE risk, suggesting the clinical utility of heart rate response testing [9]. Malik et al. (2019) demonstrated the efficacy of left ventricular mechanical dyssynchrony evaluation using phase analysis in ESRD patients, underscoring its potential as a predictive tool for adverse cardiovascular outcomes [10].

Further studies have expanded on these findings. Gowdak et al. (2012) developed new risk-scoring models to improve the prediction accuracy of MACE risk in ESRD patients undergoing kidney transplantation [11]. Feuchtner et al. (2017) showed that low-density plaque and the napkin ring sign are potent predictors of MACEs, providing new evidence for applying cardiovascular imaging in risk assessment [12]. Landray et al. (2010) discussed the validation of predictive scoring for individual ESRD and mortality, offering quantitative tools for clinical decision-making [13].

The application of machine learning in predicting MACEs shows significant potential, especially in handling large-scale electronic health record (EHR) data. Duan et al. (2019) and Betancur et al. (2017) utilized dynamic therapy information and integrated clinical and cardiovascular imaging data, respectively, demonstrating significant advantages in improving prediction accuracy and interpretability [14,15].

Blanes-Selva et al. (2021) explored machine learning techniques to predict one-year mortality in hospitalized patients. They applied five different machine learning techniques and demonstrated outstanding performance in the area under the ROC curve [16]. Heo et al. extended this work by applying machine learning to predict hidden coronary artery disease in patients with acute ischemic stroke, highlighting the broad applicability of these techniques across different medical conditions [17].

Substantial efforts have also been made to integrate machine learning with real-time data from emergency departments. Zhang et al. (2020) integrated a random forest model with hospital information systems to predict acute myocardial infarction and all-cause mortality within a month for patients with chest pain, achieving high predictive accuracy [18]. Similarly, Zheng et al. (2023) explored the feasibility of using ML to predict risk stratification within three months for patients with suspected non-ST-elevation acute coronary syndrome, demonstrating promising results [19].

Machine learning models have also been applied to specific medical procedures. Zhou et al. (2019) utilized ML to predict cardiovascular events in patients undergoing percutaneous coronary intervention, enhancing risk assessment precision [20]. Sherazi et al. (2021) introduced an ensemble classifier to predict MACEs in STEMI and NSTEMI patients, achieving high predictive accuracy over a two-year follow-up period. Deep learning models extend the predictive capabilities of ML [21]. Kim et al. (2022) developed a deep learning model to predict MACEs within one year post-discharge in acute myocardial infarction patients, showing significant predictive capability [22]. Schrempf et al. (2021) and Lin et al. (2021) highlighted the efficacy of ML techniques in predicting long-term cardiac outcomes, with robust predictive accuracy demonstrated in patients with extra-cardiac vascular diseases [23,24].

The integration of ML with EHR has been particularly effective. Liu et al. (2014) and Hu et al. (2019) leveraged EHR data to predict adverse cardiac events, enhancing predictive accuracy through advanced variable selection and evidential reasoning techniques. [25,26] Furthermore, studies by Hu et al. (2016) and Huang et al. (2017) demonstrated the potential of localized data and boosted resampling classification to predict MACEs, achieving high accuracy [27,28]. Advanced ML techniques, such as adversarial learning, have also been applied to improve predictive robustness. Huang and Dong (2018) used adversarial learning techniques on EHRs to predict MACEs in acute coronary syndrome patients, showing improved predictive robustness [29]. Moreover, Ishikita et al. (2024) highlighted the incremental value of ML in risk prediction for patients with tetralogy of Fallot [30].

ML models have also been developed for specific patient populations, such as those undergoing orthotopic liver transplantation [31] and very young patients with acute coronary syndrome [32], demonstrating high predictive accuracy for MACEs in these unique cohorts. The application of machine learning techniques in predicting major adverse cardiac events has shown remarkable effectiveness across various patient populations and clinical settings. These advancements enhance predictive accuracy, support real-time decision-making, and provide personalized patient care, ultimately improving cardiovascular health outcomes.

By systematically evaluating multiple contemporary machine learning techniques on an expanded variable set, this study aims to improve the precision of MACE risk assessments and provide actionable insights that could enhance personalized care strategies for ESRD patients. By integrating newer, more robust analytical methods, such an approach could contribute to advancing the field of clinical risk assessment.

In contrast to prior studies—many of which utilized a limited number of clinical variables and traditional machine learning models—our study incorporates a high-dimensional dataset of 84 features, including echocardiographic and radiographic markers specific to the ESRD population. While previous approaches often offered limited interpretability or clinical integration, our model combines CatBoost with SHAP analysis to ensure predictive strength and transparent, clinician-relevant insights. As shown in Table A1, our framework achieves superior AUC and clinical applicability. This work is among the first in a Taiwanese ESRD cohort to implement explainable machine learning for cardiac risk prediction, providing a practical tool for individualized risk stratification and evidence-based decision support in hemodialysis care.

## 2. Materials and Methods

### 2.1. Data Collection

The study enrolled all ESRD patients who underwent maintenance hemodialysis at the hemodialysis center in Shin Kong Wu Ho-Su Memorial Hospital, Taipei, between 1 October and 31 December 2018. The enrolled patients were followed up until death, transfer to other clinics, modality switch, or the study’s end on 31 December 2021. The primary outcomes were MACEs, which were defined as myocardial infarction, coronary revascularization, stroke, hospitalization due to heart failure, or death from cardiovascular causes.

We collected 84 variables from maintenance hemodialysis patients, including age, gender, dialysis vintage, access type and location, baseline comorbidities, lab data, medications, echocardiographic parameters, cardiothoracic ratio, and the calculation of aortic arch on chest X-ray in Table 1. Dialysis vintage and type of vascular access have been demonstrated to be associated with mortality in the population. Baseline comorbidities included DM, HTN, dyslipidemia, CAD, AMI, CVA, PAOD, HF, COPD, LC, malignancy, and arrhythmia, which impact cardiovascular events and survival [33]. Lab data including total protein (gm/dL), albumin (gm/dL), AST (IU/L), ALT (IU/L), alkaline phosphatase (IU/L) [34], total bilirubin (mg/dL), cholesterol (mg/dL), triglyceride (mg/dL), glucose (mg/dL), WBC (×1000/uL), RBC (×10^6^/µL), Hb (g/dL), Hct (%), MCV (fl), platelet (×1000/µL), Fe (µg/dL), TIBC (µg/dL), ferritin (ng/mL), transferrin saturation, aluminum (ng/mL), post-dialysis weight (kg), uric acid (mg/dL), Na (meq/L), K (meq/L) [35], iCa (mg/dL), phosphorus (mg/dL), Kt/V (Gotch), PTH (pg/mL), and Ca × P (mg^2^/dL^2^) reflect the patient’s clinical conditions such as nutritional status, dialysis efficacy, disease burden on uremia, anemia, and mineral bone disease. Medications provide vital information for the individual treatment of disease and complications. Echocardiographic parameters composed of left ventricular hypertrophy (LVH) [36,37], diastolic dysfunction, ejection fraction (EF), valve abnormalities, and inferior vena cava diameter, some of them including valve calcification, inferior vena cava diameter (IVCD), left ventricular geometry, ejection fraction, and valvular heart disease, were significantly associated with cardiovascular risks [38]. Cardiothoracic ratios (CTRs) and aortic arch calcification (AoAC) from routine chest X-rays were also risk factors for these outcomes [39]. In addition, these collected lab data and image parameters are to be routinely measured according to the KDOQI guidelines for quality control [40]. The entire table is demonstrated in Table A2.

### 2.2. Data Analysis

**Figure 1:**
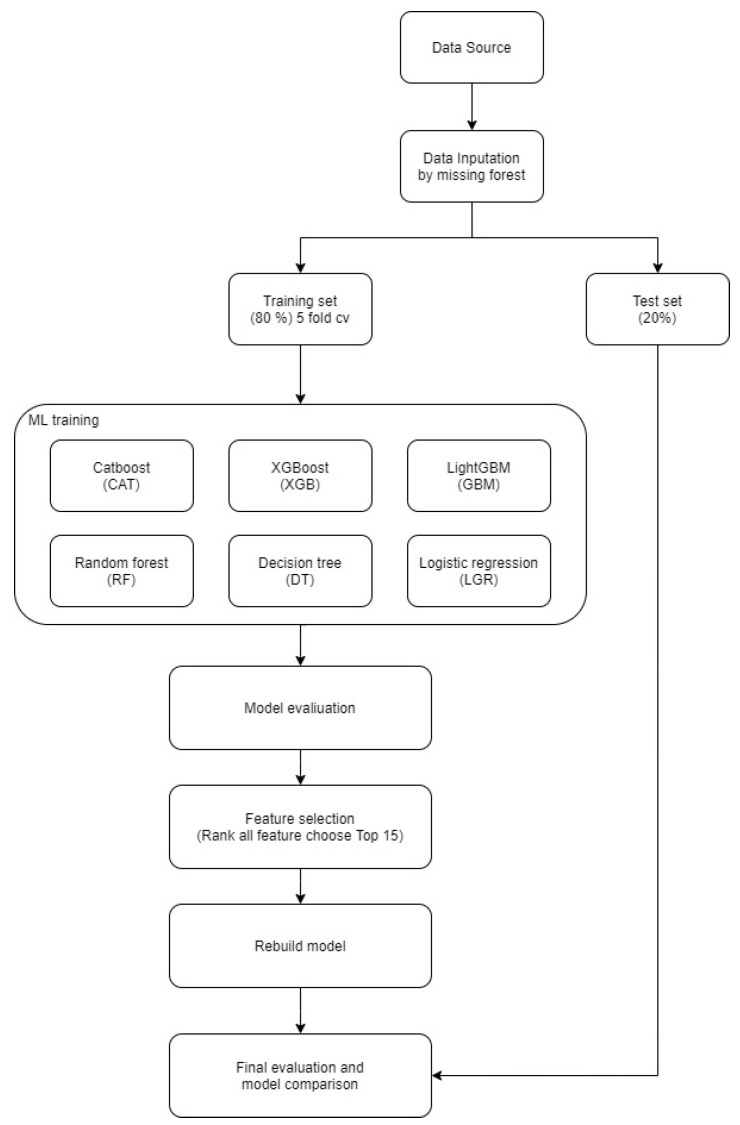
Research analysis process framework diagram.

The data analysis strategy in this study was designed to systematically compare the performance of traditional statistical approaches and advanced machine learning models in predicting MACEs in ESRD patients. As outlined in Figure 1, the analytical pipeline was implemented in sequential stages to ensure methodological rigor and clinical relevance.

Initially, raw clinical data were pre-processed, with missing values imputed using the missForest algorithm to preserve nonlinear associations among features. Categorical variables were encoded via one-hot transformation, expanding the variable set from 83 to 113 dimensions. The dataset was then stratified and split into training and testing subsets (80:20), ensuring consistent MACE prevalence across groups.

Multiple classification models—including Logistic Regression, Decision Tree, Random Forest, XGBoost, CatBoost, and LightGBM—were developed and evaluated using five-fold cross-validation. Performance metrics such as AUC, sensitivity, specificity, precision, and F1 score were calculated to assess discriminative ability.

Feature selection was performed based on model-derived importance rankings to enhance clinical applicability. The top 15 predictors identified by the CatBoost models were retained for re-modeling to improve interpretability without compromising accuracy. SHAP analysis was subsequently applied to quantify each feature’s contribution to model output, enabling a transparent, clinician-friendly interpretation of individual risk profiles.

### 2.3. Data Preparation and Imputation

Missing data are a common challenge in clinical datasets, which, if unaddressed, could bias model outcomes. To handle this, we employed the missingforest package in R, which implements a random forest-based imputation technique. This method was chosen for its proven effectiveness in handling continuous and categorical variables and its ability to capture complex interactions between variables. Compared to more straightforward imputation methods, such as mean or mode substitution, missingforest preserves the natural variability and structure of the data, making it particularly suitable for high-dimensional datasets like ours. A simulation study by Stekhoven and Bühlmann (2012) demonstrated the superior performance of missingforest in imputing missing clinical data, which further supports its adoption in this study [41].

### 2.4. Variable Encoding and Expansion

To accommodate the requirements of machine learning algorithms, categorical variables were transformed using one-hot encoding via the mltools package version 0.3.5 in R. This expanded the original 83 variables to 113, allowing models to capture subtle distinctions within categorical data better. This approach enhances model interpretability and ensures that categorical data are represented in a format compatible with gradient boosting algorithms, which perform optimally with numerical inputs.

### 2.5. Data Segmentation

To ensure an appropriate balance between model training and generalizability assessment, the dataset was divided into training and testing subsets using an 80:20 ratio, following the practical guidance proposed by Gholamy et al. (2018) [42]. Given the imbalanced nature of MACE occurrence, we applied stratified sampling to preserve the proportion of outcome classes across both subsets. This was implemented using the createDataPartition() function from the R caret package version 6.0-94, allowing for controlled stratification and reproducibility through a fixed random seed [43]. This approach ensured that model evaluation was not biased by uneven outcome distribution and provided a stable foundation for downstream cross-validation and feature analysis.

### 2.6. Model Building and Validation Phase 1

To ensure robust model evaluation, a five-fold cross-validation framework was implemented. This choice balances computational efficiency with model stability, as smaller folds may result in high variance, while larger folds could increase computational burden [42]. By averaging performance metrics across folds, this approach minimizes the risk of overfitting and provides a more reliable assessment of each model’s predictive capabilities.

Hyperparameter tuning for the ensemble models (XGBoost, CatBoost, and LightGBM) was conducted using GridSearchCV within a five-fold cross-validation framework to identify optimal configurations. Appendix C, Table A3, Table A4 and Table A5 provide a detailed summary of the selected parameters.

### 2.7. Model Refinement and Variable Selection Phase 2

AUC is a critical metric for evaluating a model’s overall performance, especially in binary classification tasks. Based on the categorization established by Hosmer Jr, Lemeshow, and Sturdivant (2013), as well as Nassar Jr et al. (2012), AUC values are divided into several distinct levels: values between 0.5 and 0.6 are considered poor, 0.6 to 0.7 are moderate, 0.7 to 0.8 are good, 0.8 to 0.9 are very good, and those above 0.9 are classified as excellent [44,45].

In the second phase of our study, we implemented an AUC threshold of 0.6 to guide the selection of models for further analysis. Models that surpassed this threshold underwent re-modeling for a more comprehensive performance evaluation. During this phase, we focused on the top-performing model from Phase 1, scoring thoroughly and ranking all variables based on their feature importance to the model’s predictive accuracy.

The top 15 variables identified from this process were then used for re-modeling, enabling a more concentrated comparison of their impact on MACEs. Using feature selection techniques, the original high-dimensional dataset was reduced to smaller dimensions, effectively enhancing the prediction and evaluation of MACEs in patients. These 15 variables represent the most significant factors influencing the occurrence of MACEs.

We employed SHAP (SHapley Additive exPlanations) to enhance model interpretability and clinical applicability, transforming complex machine learning outputs into interpretable feature contributions [46]. SHAP’s ability to rank predictors by their impact on model outcomes enables clinicians to focus on critical risk factors, optimize diagnostic workflows, and personalize treatment strategies, particularly in high-pressure settings like critical care [47]. Positive SHAP values highlight increased risk and guiding interventions, while negative values reflect mitigative factors, reinforcing effective treatments [48]. Its intuitive visualizations improve doctor–patient communication, enhancing patient understanding and adherence to medical recommendations.

For clinical integration, the SHAP workflow involves generating summary plots to identify key predictors, translating insights into actionable plans by addressing high-risk factors or monitoring protective ones, and implementing regular SHAP updates. These updates allow dynamic decision-making, support precision medicine, and improve healthcare outcomes.

## 3. Results

This section presents the findings from the machine learning models applied to the ESRD dataset, including performance metrics, feature importance analysis, and the interpretation of the predictive variables associated with MACEs. The results are organized by model type and supported by SHAP-based explainability techniques to enhance clinical interpretability.

### 3.1. Phase 1

**Figure 2:**
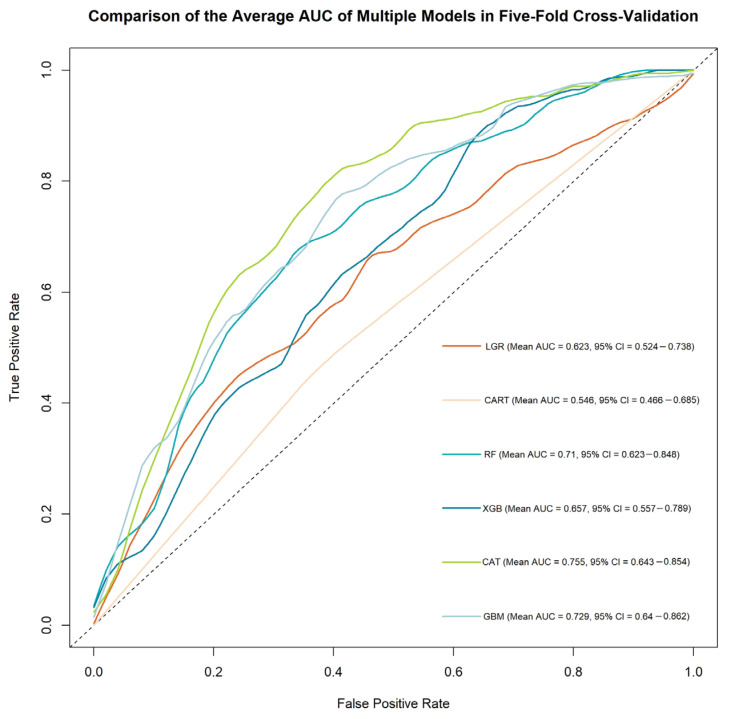
Comparison of ROC Curves and AUCs for ML models in MACE prediction.

This study employed various evaluation metrics to compare six machine learning models: Logistic Regression, Decision Tree, Random Forest, XGBoost, CatBoost, and LightGBM. The performance metrics for each model are summarized in Table 2, with the corresponding ROC curves displayed in Figure 2.

CatBoost demonstrated the best overall performance, achieving an accuracy of 72.8% and a Kappa coefficient of 0.456, highlighting its superior predictive accuracy and consistency. Conversely, the Decision Tree exhibited the weakest performance, with an accuracy of 55.8% and a Kappa coefficient of 0.092. Regarding sensitivity and specificity, XGBoost achieved the highest sensitivity of 0.829, indicating its effectiveness in identifying positive cases. However, its specificity was significantly lower at 0.492, suggesting a tendency to overfit on positive cases. In contrast, Random Forest and LightGBM displayed more balanced performances, with specificity exceeding 0.73 in both models.

Using AUC as a threshold to evaluate model discrimination, CatBoost and LightGBM attained AUC values above 0.72, indicating good predictive power. The Decision Tree performed notably worse, with an AUC of 0.546, underscoring its limited capability in high-dimensional datasets.

CatBoost’s superior performance can be attributed to its unique handling of categorical features and its gradient-boosting technique, which minimizes overfitting and enhances generalization. Meanwhile, XGBoost’s high sensitivity emphasizes its focus on positive cases, albeit at the cost of specificity. Table 2 provides a comprehensive comparison of all models.

Table 3 shows the confusion matrix of the CatBoost model during five-fold cross-validation, illustrating its practical predictive distribution. The model accurately identified 29 of 34 MACE cases and 33 of 48 non-MACE cases, resulting in a balanced performance suitable for further model refinement.

### 3.2. Phase 2

#### Feature Selection and Model Refinement

**Figure 3:**
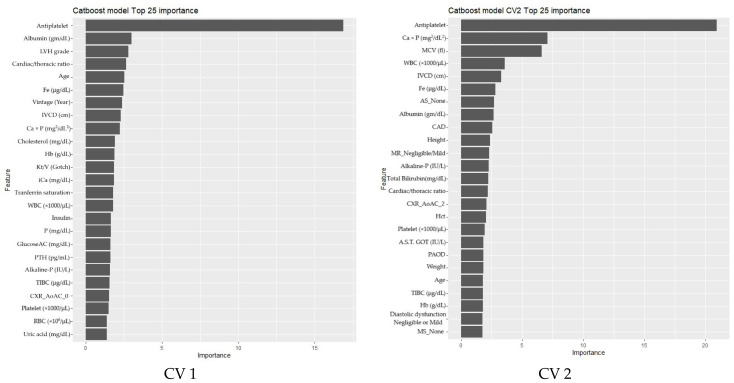

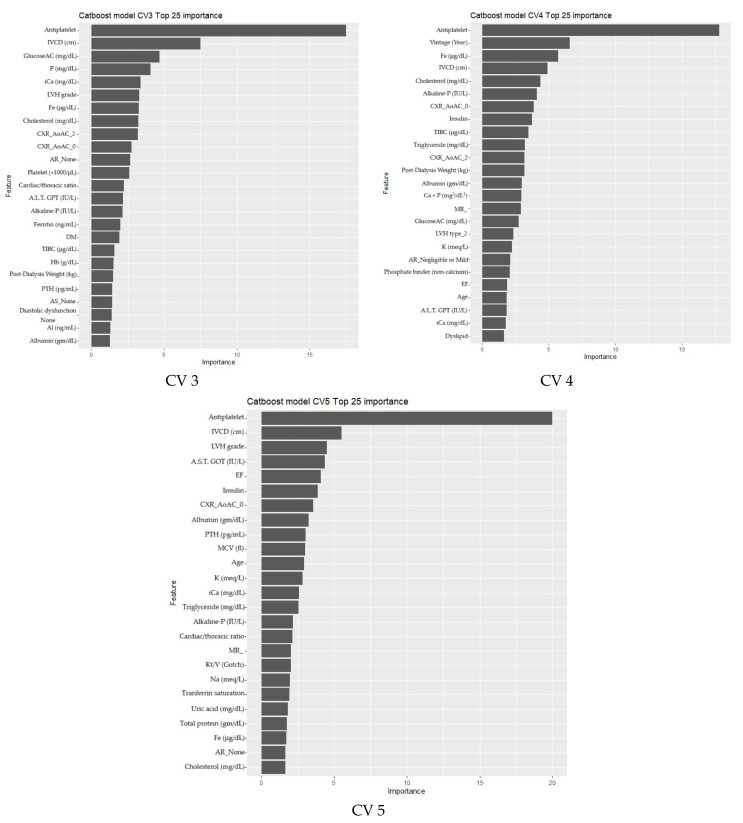
CatBoost five-fold cross-validation result: feature importance plots. Note: the bar plots depict the top features for each cross-validation fold. Consistency in feature rankings across folds indicates CatBoost’s robustness in identifying critical predictors for MACEs in ESRD patients.

Based on the initial modeling results, the CatBoost model demonstrated consistently superior performance across all cross-validation folds. Figure 3 illustrates the feature importance rankings derived from the CatBoost model during five-fold cross-validation. Throughout all folds, several features, including “Antiplatelet”, “LVH grade”, “Age”, and “Albumin”, consistently ranked among the top predictors, underscoring their critical contribution to MACE prediction in ESRD patients.

The stability of feature rankings across folds highlights the reliability of the CatBoost model in identifying clinically meaningful predictors. For instance, antiplatelet therapy consistently emerged as a top feature, reflecting its vital role in mitigating thrombotic risks in hemodialysis patients. LVH grade showed high importance, emphasizing the significance of left ventricular hypertrophy as a key cardiovascular risk factor in this population. Albumin and age were also notable, aligning with their established roles in reflecting nutritional status and demographic influences on cardiovascular outcomes.

These findings confirmed the robustness of the top 15 features identified (presented in Table 4), which were subsequently employed for re-modeling and further evaluation. Leveraging these reduced dimensions resulted in a minimal loss of predictive performance while improving interpretability.

### 3.3. Model Evaluation Summary

**Figure 4:**
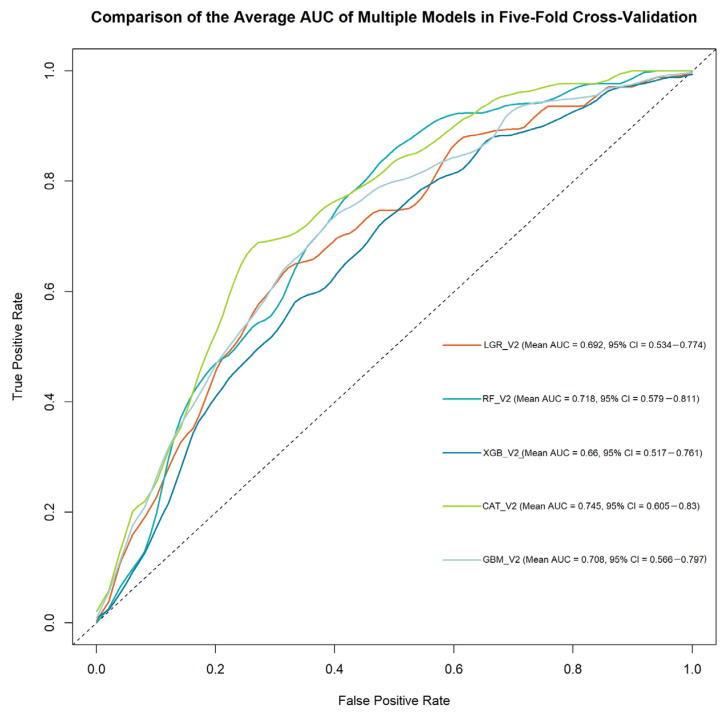
Comparison of ROC curves and AUCs for rebuilding ML models in MACE prediction.

In Phase 2, re-modeling was conducted using the top 15 features identified through model-driven importance analysis. The evaluation metrics for all five machine learning models are summarized in Table 5, with corresponding ROC curves shown in Figure 4.

CatBoost demonstrated the most balanced and clinically meaningful performance, with an AUC of 0.745 (95% CI: 0.605–0.830), an accuracy of 72.4%, and the highest F1 score (0.810) among all models. This indicates the model’s strong capacity to optimize sensitivity and precision, an important consideration in clinical screening where false positives and negatives carry substantial consequences. The associated confusion matrix (Table 6) shows that CatBoost correctly classified 27 out of 34 MACE cases while maintaining high specificity by correctly identifying 37 out of 48 non-MACE cases.

XGBoost yielded the highest sensitivity (0.829), reaffirming its ability to detect true positives. However, its specificity remained limited (0.492), suggesting a tendency toward overprediction of MACEs. Random Forest, with an AUC of 0.718 (95% CI: 0.579–0.811) and sensitivity of 0.800, offered a compromise between detection capability and overall accuracy. LightGBM provided relatively stable results across all metrics (0.708 AUC, 0.637 specificity, and 0.729 sensitivity) but trailed CatBoost in both precision and F1 score. Logistic Regression, while providing a reliable reference model (0.692 AUC), was consistently outperformed by ensemble methods in key metrics.

These findings support the value of ensemble boosting algorithms—particularly CatBoost—for structured clinical data settings, where interpretability, class imbalance, and moderate sample size are relevant challenges. Notably, the improvement in AUC and F1 score compared to Phase 1 further highlights the utility of model-guided feature selection.

**Figure 5:**
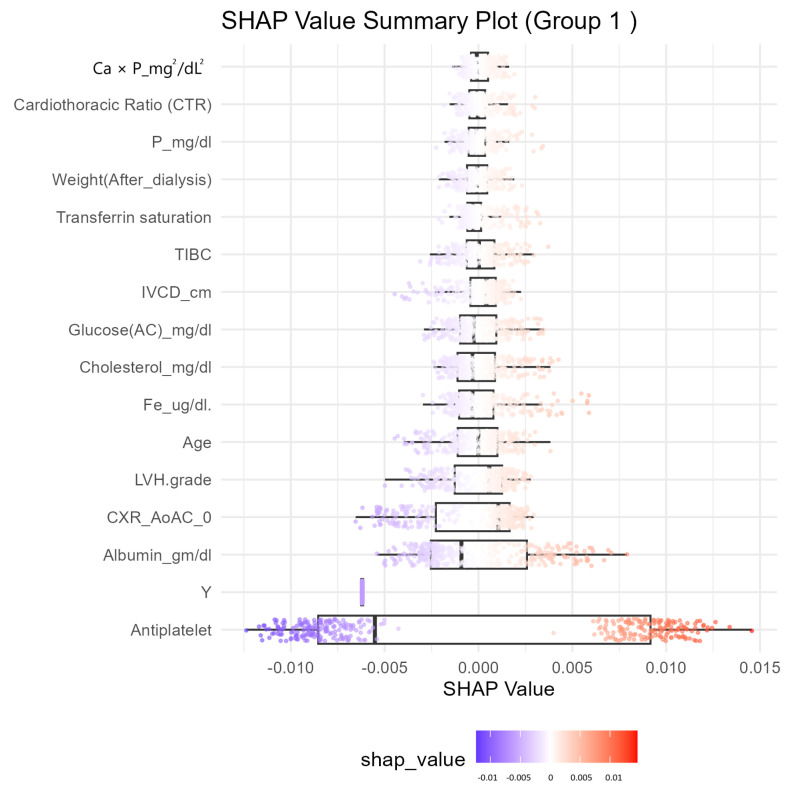
CatBoost cross-validation SHAP value summary plot.

In Figure 5, the SHAP analysis of the CatBoost model revealed key predictors of MACEs in ESRD patients, offering interpretability and actionable insights for clinical decision-making. Antiplatelet therapy was identified as the most significant factor, with positive SHAP values indicating a strong association with increased risk. Left ventricular hypertrophy (LVH) grade and age also contributed significantly, reinforcing their established roles as cardiovascular risk factors. Conversely, higher albumin levels showed negative SHAP values, suggesting a protective effect. Simultaneously, the cardiothoracic ratio (CTR) and markers of mineral metabolism, such as the calcium-phosphate product (Ca × P) and transferrin saturation, further highlighted the multifactorial nature of MACE risk in ESRD.

SHAP values provided a clear direction for interventions, prioritizing features with high positive contributions, such as antiplatelet therapy and LVH grade, while emphasizing the importance of maintaining protective factors like albumin. Intuitive visualizations enhanced understanding and facilitated patient-clinician communication, improving adherence to treatment plans. By translating complex model outputs into actionable insights, SHAP reinforced the clinical relevance and utility of machine learning in precision medicine. These findings underscore the potential of SHAP analysis in guiding personalized risk stratification and optimizing care strategies for ESRD patients, bridging the gap between advanced predictive modeling and practical clinical application.

## 4. Discussion

The outstanding performance of the CatBoost model, particularly in accuracy, precision, F1 score, and AUC, highlights its high reliability and effectiveness in classification tasks. Random Forest and LightGBM also perform strongly, especially in identifying true positives. However, despite its excellent sensitivity, XGBoost exhibits potential shortcomings in accurately identifying negative cases. These results emphasize the importance of selecting appropriate models based on specific clinical task requirements and metric priorities. XGBoost may be more suitable for tasks that prioritize identifying positive cases. At the same time, the balanced performance of CatBoost makes it ideal for applications that require high overall prediction accuracy and reliability.

### 4.1. Clinical Implications

The clinical relevance of our findings lies not only in the model’s predictive capacity but, more importantly, in identifying risk markers that are both data-driven and physiologically plausible in the ESRD population. Among the top-ranked features, antiplatelet use and LVH grade emerged as consistent predictors of adverse cardiac outcomes. This observation aligns with well-established clinical concerns: LVH is a surrogate for chronic pressure overload and structural remodeling in dialysis patients, and its presence often signals subclinical cardiac compromise. Likewise, antiplatelet therapy is frequently initiated in high-risk individuals but may also reflect a pre-existing atherosclerotic burden, which inherently elevates MACE risk.

From a practical standpoint, integrating these variables into a clinical decision support framework may enhance risk stratification at key time points, such as preoperative cardiovascular evaluation or dialysis initiation. A tool based on the current model could assist clinicians in identifying patients who warrant more intensive cardiology surveillance, echocardiographic follow-up, or medication review. For example, patients with moderate-to-severe LVH on echocardiograms, even in the absence of overt symptoms, may benefit from tailored cardioprotective interventions.

While respectable, an AUC of 0.745 (95% CI: 0.605–0.830) does not alone establish readiness for direct clinical deployment. The model’s performance should be viewed as a starting point for iterative refinement rather than an endpoint. Compared to other published models—such as the deep learning approach by Kim et al. (AUC 0.76) [22] or the ensemble classifier by Sherazi et al. (AUC 0.78) [21]—our model demonstrates competitive accuracy within an ESRD-specific context, but its value lies in interpretability and domain alignment rather than numerical superiority alone. The model, built using clinically curated features accessible within routine care that can be explained using SHAP values, supports its potential as a clinically meaningful adjunct to existing practice—not a replacement.

Importantly, any predictive model in nephrology must meet a higher standard for trust and transparency, given the complexity and fragility of dialysis populations. Prospective validation, user-centered interface design, and close collaboration with frontline nephrologists will be essential before such tools are introduced into routine care workflows.

### 4.2. Limitations and Generalizability

This analysis was conducted using data from a single medical center, which inevitably introduces constraints on generalizability. While internal validation demonstrated consistent performance across cross-validation folds, applying the model to external ESRD populations with differing referral patterns, treatment protocols, or demographic characteristics remains essential for verifying its broader applicability.

The model was trained on a curated dataset with predefined inclusion criteria, which may have excluded patients with incomplete records or atypical clinical profiles. Although imputation methods were applied to reduce information loss, selection bias cannot be entirely ruled out. This limitation is inherent in most retrospective observational designs, particularly those involving electronic health record data.

One crucial methodological limitation is the absence of electrocardiographic (ECG) variables. Prior studies have established that ECG-derived features—such as QTc prolongation, T-wave alternans, and intraventricular conduction delays—predict adverse cardiac outcomes in dialysis patients. However, in our cohort, ECG data were not consistently available in a structured format and could not be reliably extracted for analysis. The potential prognostic contribution of these variables is acknowledged, and future model iterations should aim to incorporate structured or signal-based ECG data where feasible.

The scope of model comparison was limited to selected ensemble and traditional classifiers. This decision was based on the relatively modest sample size and the need for interpretability in a clinical context. While this approach aligns with the model’s intended use in practice, it restricts direct comparison with more complex architectures, such as deep neural networks. Lastly, although we employed SHAP values to support interpretability, the clinical integration of these explanations has not been evaluated and should be addressed in future implementation studies.

### 4.3. Future Directions

The results of this study highlight the potential of machine learning methods in medical research, particularly in managing high-dimensional, nonlinear, and complex data interactions. Although many machine learning algorithms are available, we deliberately selected ensemble methods—CatBoost, XGBoost, and LightGBM—along with traditional models as benchmarks due to their proven performance in similar clinical applications and the necessity for interpretable results. As larger and more diverse datasets become available in future studies, we plan to investigate deep learning and reinforcement learning approaches, which may improve predictive performance and provide additional insights into ESRD patient data. Moreover, integrating multidimensional omics data, including genomics, proteomics, and metabolomics, represents a promising avenue for thoroughly understanding the pathological mechanisms of ESRD, leading to more effective treatment strategies.

From a translational perspective, future research should also explore the real-world implementation of machine learning models in clinical settings. This includes tackling technical barriers, such as integration with EHR systems, user interface design, and regulatory compliance. In addition, long-term studies assessing the impact of these models on patient outcomes, cost-effectiveness, and healthcare delivery will be crucial to demonstrating their practical value.

## 5. Conclusions

This study offers a data-driven approach for selecting variables that significantly impact the prognosis of ESRD patients. It establishes a foundation for the broader application of machine learning techniques in nephrology research. By tackling clinical challenges such as personalized risk assessment and treatment optimization, these methods can enhance the quality of care for ESRD patients. With the ongoing advancement of data science and artificial intelligence technologies, we expect to achieve breakthrough discoveries that will ultimately provide ESRD patients with more personalized and precise medical services.

## Figures and Tables

**Table 1:** Initial demographic and clinical profiles of the research cohort.

	Overall (*n* = 412)	MACE		*p*-Value
		Never Occurred (*n* = 242)	Occurred (*n* = 170)	
Age (mean (SD))	69.19 (12.14)	67.96 (12.59)	70.94 (11.29)	0.014
Gender = Female (%)	192 (46.6)	122 (50.4)	70 (41.2)	0.08
Vintage (Year) (mean (SD))	8.18 (7.25)	8.83 (7.81)	7.25 (6.29)	0.029
AV cal (%)	196 (62.6)	106 (56.1)	90 (72.6)	0.005
AS (%)				0.029
-	101 (24.5)	56 (23.1)	45 (26.5)	
None	277 (67.2)	172 (71.1)	105 (61.8)	
Negligible or Mild	22 (5.3)	12 (5.0)	10 (5.9)	
Moderate	11 (2.7)	2 (0.8)	9 (5.3)	
Severe	1 (0.2)	0 (0.0)	1 (0.6)	
LVH (%)	237 (79.3)	135 (74.6)	102 (86.4)	0.02
LVH grade (mean (SD))	1.97 (1.23)	1.82 (1.27)	2.21 (1.13)	0.007
LVH type (%)				0.024
1	131 (43.8)	72 (39.8)	59 (50.0)	
2	106 (35.5)	63 (34.8)	43 (36.4)	
3	28 (9.4)	24 (13.3)	4 (3.4)	
4	34 (11.4)	22 (12.2)	12 (10.2)	
EF(%) (mean (SD))	67.20 (12.10)	68.65 (11.16)	65.04 (13.13)	0.012
IVCD (cm) (mean (SD))	1.50 (0.43)	1.44 (0.43)	1.62 (0.41)	0.003
Comorbidities				
DM (%)	198 (48.1)	99 (40.9)	99 (58.2)	0.001
CAD (%)	173 (42.0)	87 (36.0)	86 (50.6)	0.004
AMI (%)	12 (2.9)	3 (1.2)	9 (5.3)	0.035
PAOD (%)	111 (26.9)	53 (21.9)	58 (34.1)	0.008
HF (%)	91 (22.1)	44 (18.2)	47 (27.6)	0.031
Amputation (%)				0.026
0	405 (98.3)	241 (99.6)	164 (96.5)	
1.1	2 (0.5)	1 (0.4)	1 (0.6)	
1.2	5 (1.2)	0 (0.0)	5 (2.9)	
Albumin (gm/dL) (mean (SD))	3.86 (0.38)	3.92 (0.33)	3.78 (0.42)	<0.001
Hb (g/dL) (mean (SD))	10.38 (1.44)	10.50 (1.44)	10.19 (1.42)	0.029
Ca × P (mg^2^/dL^2^) (mean (SD))	46.88 (13.21)	45.81 (12.66)	48.45 (13.86)	0.048
CXR_AoAC (%)				<0.001
0	120 (31.4)	90 (40.5)	30 (18.8)	
1	83 (21.7)	49 (22.1)	34 (21.2)	
2	107 (28.0)	49 (22.1)	58 (36.2)	
3	72 (18.8)	34 (15.3)	38 (23.8)	
Cardiac/thoracic ratio (%) (mean (SD))	0.52 (0.07)	0.51 (0.06)	0.53 (0.07)	<0.001
Medication				
alpha-glucose inhibitor (%)	11 (2.7)	1 (0.4)	10 (5.9)	0.002
Insulin (%)	85 (20.6)	33 (13.6)	52 (30.6)	<0.001
RI/ACEI/ARB (%)	217 (52.7)	117 (48.3)	100 (58.8)	0.046
Antiplatelet (%)	199 (48.3)	82 (33.9)	117 (68.8)	<0.001

Note: AV cal = aortic valve calculation; AS = aortic stenosis; LVH = left ventricular hypertrophy; EF = ejection fraction; IVCD = inferior vena cava diameter; DM = diabetes mellitus; CAD = coronary artery disease; AMI = acute myocardial infarction; PAOD = peripheral arterial occlusion Disease; HF = heart failure; Hb = hemoglobin; Ca × P = calcium-phosphate product in blood; CXR_AoAC = chest X-ray for aortic arch calcification; RI/ACEI/ARB = renin inhibitor/angiotensin converting enzyme inhibitors/angiotensin receptor blocker.

**Table 2:** Evaluation of initial modeling for predicting MACEs in ESRD patients.

ML Method	Accuracy	Kappa	Sensitivity	Specificity	Precision	F1	AUC (95% CI)
LGR	0.633	0.264	0.635	0.631	0.569	0.584	0.623 (0.524–0.738)
CART	0.558	0.092	0.476	0.616	0.465	0.47	0.546 (0.466–0.685)
RF	0.706	0.399	0.665	0.735	0.648	0.652	0.71 (0.623–0.848)
XGB	0.626	0.285	0.812	0.496	0.536	0.638	0.657 (0.557–0.789)
CatB	0.728	0.456	0.765	0.703	0.651	0.7	0.755 (0.643–0.854)
lightGBM	0.711	0.41	0.671	0.74	0.672	0.655	0.729 (0.64–0.862)

Note: LGR = Logistic Regression; CART = Decision Tree; RF = Random Forest; XGB = XGBoost; CatB = CatBoost.

**Table 3:** Confusion matrix from Phase 1: the best CatBoost model during cross-validation.

		Reference	
		Non-MACE	MACE
Prediction	Non-MACE	33	5
	MACE	15	29

**Table 4:** The top 15 variables ranked by importance after five-fold cross-validation.

Ranking	Feature	Score
1	Antiplatelet	565
2	LVH.grade	542
3	CXR_AoAC_0	531
4	IVCD (cm)	526
5	Age	518
6	P (mg/dL)	506
7	Albumin (gm/dL)	503
8	GlucoseAC (mg/dL)	498
9	Cholesterol (mg/dL)	497
10	Ca × P (mg^2^/dL^2^)	490
11	Cardiac/thoracic ratio (%)	486
12	Tranferrin saturation (%)	484
13	Fe (**μ**g/dL)	479
14	TIBC (**μ**g/dL)	472
15	Post-Dialysis Weight (kg)	468

Note: The score calculation method is as follows: In the five-fold cross-validation, the fields are sorted according to their importance, with the highest being 113 and the lowest 1. The scores from the five folds are then summed up to obtain the final score.

**Table 5:** Evaluation of remodeling for predicting MACEs in ESRD Patients.

ML Method	Accuracy	Kappa	Sensitivity	Specificity	Precision	F1	AUC (95% CI)
LGR	0.654	0.340	0.824	0.537	0.570	0.667	0.692 (0.534–0.774)
RF	0.692	0.398	0.8	0.616	0.612	0.682	0.718 (0.579–0.811)
XGB	0.631	0.299	**0.829**	0.492	0.539	0.651	0.660 (0.517–0.761)
CatB	**0.724**	**0.438**	0.718	**0.728**	**0.65**	**0.81**	**0.745 (0.605–0.830)**
lightGBM	0.675	0.353	0.729	0.637	0.586	0.649	0.708 (0.566–0.797)

Note: LGR = Logistic Regression; RF = Random Forest; XGB = XGBoost; CatB = CatBoost.

**Table 6:** Confusion matrix from Phase 2: the best CatBoost model during cross-validation.

		Reference	
		Non-MACE	MACE
Prediction	Non-MACE	37	7
	MACE	11	27

## Data Availability

The datasets presented in this article are not readily available because they consist of patient-specific medical data obtained from hospital records, and access requires explicit approval from the hospital administration and joint application with collaborating researchers. Requests to access the datasets should be directed to the co-first author.

## Abbreviations

The following abbreviations are used in this manuscript:

LGR	Logistic Regression
CART	Decision Tree
SVM	Support Vector Machine
RF	Random Forest
XGB	XGBoost
CatB	CatBoost
NB	Naive Bayes
ANN	Artificial neural network
DNN	Deep neural networks
KNN	K-Nearest Neighbor
ESRD	End-stage renal disease
MACE	Major adverse cardiac event
SHAP	SHapley Additive exPlanations
AUC	Area under receiver operator characteristic curve
AV cal	Aortic valve calculation
AS	Aortic stenosis
LVH	Left ventricular hypertrophy
EF	Ejection fraction
IVCD	Inferior vena cava diameter
DM	Diabetes mellitus
CAD	Coronary artery disease
AMI	Acute myocardial infarction
PAOD	Peripheral arterial occlusion disease
HF	Heart failure
Hb	Hemoglobin
Ca × P	Calcium-phosphate product in blood
CXR_AoAC	Chest X-ray for aortic arch calcification
CTR	Cardiacthoracic ratio
RI/ACEI/ARB	Renin inhibitor/angiotensin-converting enzyme inhibitors/angiotensin receptor blocker
EHR	Electronic health record
ECG	Electrocardiogram

## Appendix A

**Table A1:** Summary of machine learning-based studies for cardiovascular event prediction.

Study	Dataset	Methodology	Proposed Model	Evaluation Metrics	Results
A machine learning-based approach for predicting the outbreak of cardiovascular diseases in patients on dialysis.	Italian dataset (Istituto di Fisiologia Clinica); American dataset (NIDDK repository).	SVM with RBF kernel, optimized using GridSearch.	Nonlinear SVC with RBF kernel.	Accuracy	95.25% (Italian); 92.15% (American).
Utilizing dynamic treatment information for MACE prediction of acute coronary syndrome.	2930 ACS patient samples with 232 static and 2194 dynamic features.	Bidirectional recurrent neural network (RNN) on EHR data.	Dynamic, Boosted-RMTM RNN-based deep learning model.	AUC Accuracy	0.713. 0.764.
Rest scan does not improve automatic machine learning prediction of major adverse coronary events after high-speed myocardial perfusion imaging.	2619 patients undergoing high-speed SPECT imaging.	Machine learning using clinical and imaging variables from stress and rest scans.	Not mentioned.	AUC	0.81.
Design of 1-year mortality forecast at hospital admission: a machine learning approach.	Retrospective dataset from EHR from Hospital La Fe with 36 features.	SVM, KNN, gradient boosting, RF, and multilayer perceptron.	Gradient boosting classifier (best performer).	AUC	0.911.
				Sensitivity	0.858.
				Specificity	0.807.
Prediction of hidden coronary artery disease using machine learning in patients with acute ischemic stroke.	1710 patients in training set, 348 in validation.	Build 2 models for any CAD and obstructive CAD.	XGB (any CAD); LGR (obstructive CAD).	AUC	0.763; 0.714.
Real-time AI prediction for major adverse cardiac events in emergency department patients with chest pain.	85,254 ED patients with chest pain from 3 hospitals.	RF, LGR, SVC, and KNN.	RF model integrated with HIS.	AUC	AMI < 1 mo: (0.915); mortality < 1 mo (0.999).
Exploring the feasibility of machine learning to predict risk stratification within 3 months in chest pain patients with suspected NSTE-ACS.	NSTE-ACS chest pain patients from Beijing Anzhen Emergency Chest Pain Center and Beijing Bo’ai Hospital.	Five classifiers including NB, LGR, Linear SVC, etc.	NB, LGR, and Linear SVC.	AUC	0.88–0.98.
				Accuracy	0.8–1.
				Precision	0.8–1.
				Recall	0.8–1.
				F-measure	0.8–1.
Machine learning-based cardiovascular event prediction for percutaneous coronary intervention.	986 PCI patients (clinical features).	XGB, LightGBM, NN, and SVM.	LightGBM.	AUC	0.73
				F1 Score	0.86.
A soft voting ensemble classifier for early prediction and diagnosis of occurrences of major adverse cardiovascular events for STEMI and NSTEMI.	Korea Acute Myocardial Infarction Registry (KAMIR) (11,189 patient).	Soft voting ensemble combining RF, Extra Tree, gradient boosting, and SVE with five-fold cv.	Soft voting ensemble (SVE).	AUC	0.994–0.996
				Accuracy	0.888–0.909
Deep learning-based prediction model of occurrences of major adverse cardiac events during 1-year follow-up after hospital discharge in patients with AMI using knowledge mining.	Korea Acute Myocardial Infarction Registry; 10,813 AMI patients.	DNN, GBM, and GLM; hyperparameter tuning via grid search.	DNN.	AUC	0.94–0.96.
Machine learning-based risk prediction for major adverse cardiovascular events.	128,000 admissions of 29,262 patients were included in the MACE group.	GLM, RF, GBM, and LDA hyperparameter tuning via grid search.	RF (best performer).	AUC	0.88.
Machine learning to predict long-term cardiac-relative prognosis in patients with extra-cardiac vascular disease.	636 patients with IS, TIA, and/or PAD; clinical and CCTA data.	ML with automated feature selection; compared to MDI, SIS, SSS, and FRS.	Unnamed ML model.	AUC	0.92.
Prediction of adverse cardiac events in emergency department patients with chest pain using machine learning for variable selection	702 patients with non-traumatic chest pain at Singapore ED	RF for feature selection, geometric distance-based ML scoring	ML scoring model	AUC	3 variables: 0.812.
				Sensitivity	0.828.
				Specificity	0.634.
Evidential MACE prediction of acute coronary syndrome using electronic health records.	2930 ACS patient samples (clinical EHR data).	Hybrid model using Rough Set Theory (RST) + Dempster–Shafer Theory (DST) integrating ML outputs.	Evidential Ensemble Model (RST + DST).	AUC:	0.715.
Utilizing Chinese admission records for MACE prediction of acute coronary syndrome.	2930 ACS patient admission records from a Chinese hospital.	Hybrid of rule-based NLP + CRFs for feature extraction; ML classifiers applied SVM, RF, NB, and L1-LGR.	RF (best performer).	AUC	0.72.
MACE prediction of acute coronary syndrome via boosted resampling classification using electronic medical records.	2930 ACS patient EMRs with 268 features.	Boosting with over-sampling and under-sampling to balance MACE data.	Boosted resampling classifier.	AUC	0.672.
Incremental value of machine learning for risk prediction in tetralogy of Fallot.	25 rTOF patient reviews by 5 ACHD experts.	Comparison between ML and expert clinical risk scoring.	Unnamed ML model.	AUC	AGE ≥ 20 0.85; AGE < 20 0.98.
Machine learning models to predict major adverse cardiovascular events after orthotopic liver transplantation: a cohort study.	1459 OLT patients.	RF, SVM, XGB, and LGR.	XGB (best performer).	AUC	0.71 (0.63–0.79).
Using machine learning techniques to predict MACE in very young acute coronary syndrome patients.	492 patients aged under 40 with coronary angiography.	Machine learning models (SVM, NB, MLP, LDA, RF, LassoLGR, L1LGR, and L2LGR).	RF (best performer).	AUC	0.79 (0.69–0.88).
A stacking ensemble prediction model for the occurrences of major adverse cardiovascular events in patients with acute coronary syndrome on imbalanced data.	Korea Acute Myocardial Infarction Registry (KAMIR-NIH).	7 ML models (LGR, SVM, KNN, DT, RF, XGBt, and AdaB) as base learners + stacking ensemble.	Stacking ensemble with SMOTETomek.	AUC	0.9863.
				Accuracy	0.9862.
				F1-score	0.9862.
A Deep-Learning Neural Network-Based Predictive System for the Occurrence of Major Adverse Cardiovascular Events (MACE) in Patients with Acute Myocardial Infarction	KAMIR-IV dataset, 11,189 AMI patients	DNN, SMOTE for data imbalance, hyperparameter tuning via grid search	DNN-based prediction system	Accuracy	0.9835.
				AUC	0.9943.
Machine learning for major adverse cardiac events prediction in patients with acute coronary syndrome: ADDICT-ICCU study.	ADDICT-ICCU registry (1499 ACS patients; 39 centers in France).	XGB for feature selection; RF for prediction; compared to TIMI, GRACE, and traditional models.	ML-based model (XGB + RF).	AUC	0.96.
Machine learning for early prediction of major adverse cardiovascular events after first percutaneous coronary intervention in patients With AMI.	1531 AMI patients post-PCI; 1362 patients followed up.	ANN, KNN, SVM, RF, and LGR 7-fold CV.	ANN.	AUC	0.8049.
				Accuracy	0.8052.
				F1-score	0.7947.

## Appendix B

**Table A2:** Comprehensive patient demographic information table.

	Overall (*n* = 412)	MACE		*p*-Value
		Never Occurred (*n* = 242)	Occurred (*n* = 170)	
Age (mean (SD))	69.19 (12.14)	67.96 (12.59)	70.94 (11.29)	0.014
Gender = Female (%)	192 (46.6)	122 (50.4)	70 (41.2)	0.08
Vintage (Year) (mean (SD))	8.18 (7.25)	8.83 (7.81)	7.25 (6.29)	0.029
Height (mean (SD))	161.76 (8.51)	161.84 (8.86)	161.65 (8.02)	0.832
Weight (mean (SD))	60.13 (13.27)	60.47 (13.51)	59.66 (12.95)	0.54
AVA = Artificial Fistula (%)	57 (13.8)	31 (12.8)	26 (15.3)	0.566
Location of AVA (%)				0.837
Left Forearm	306 (74.3)	177 (73.1)	129 (75.9)	
Left Upper Arm	56 (13.6)	36 (14.9)	20 (11.8)	
Right Forearm	34 (8.3)	20 (8.3)	14 (8.2)	
Right Upper Arm	16 (3.9)	9 (3.7)	7 (4.1)	
AV cal (%)	196 (62.6)	106 (56.1)	90 (72.6)	0.005
AR (%)				0.142
-	101 (24.5)	56 (23.1)	45 (26.5)	
None	178 (43.2)	115 (47.5)	63 (37.1)	
Negligible or Mild	125 (30.3)	68 (28.1)	57 (33.5)	
Moderate	8 (1.9)	3 (1.2)	5 (2.9)	
AS (%)				0.029
-	101 (24.5)	56 (23.1)	45 (26.5)	
None	277 (67.2)	172 (71.1)	105 (61.8)	
Negligible or Mild	22 (5.3)	12 (5.0)	10 (5.9)	
Moderate	11 (2.7)	2 (0.8)	9 (5.3)	
Severe	1 (0.2)	0 (0.0)	1 (0.6)	
MV cal = 1 (%)	125 (39.9)	67 (35.4)	58 (46.8)	0.06
MR (%)				0.117
-	101 (24.5)	56 (23.1)	45 (26.5)	
None	62 (15.0)	44 (18.2)	18 (10.6)	
Negligible or Mild	194 (47.1)	116 (47.9)	78 (45.9)	
Moderate	46 (11.2)	22 (9.1)	24 (14.1)	
Severe	9 (2.2)	4 (1.7)	5 (2.9)	
MS (%)				0.737
-	101 (24.5)	56 (23.1)	45 (26.5)	
None	305 (74.0)	182 (75.2)	123 (72.4)	
Negligible or Mild	5 (1.2)	3 (1.2)	2 (1.2)	
Moderate	1 (0.2)	1 (0.4)	0 (0.0)	
Diastolic dysfunction (%)				0.628
-	102 (24.8)	56 (23.1)	46 (27.1)	
None	187 (45.4)	109 (45.0)	78 (45.9)	
Negligible or Mild	106 (25.7)	66 (27.3)	40 (23.5)	
Moderate	15 (3.6)	9 (3.7)	6 (3.5)	
Severe	2 (0.5)	2 (0.8)	0 (0.0)	
LVH (%)	237 (79.3)	135 (74.6)	102 (86.4)	0.02
LVH grade (mean (SD))	1.97 (1.23)	1.82 (1.27)	2.21 (1.13)	0.007
LVH type (%)				0.024
1	131 (43.8)	72 (39.8)	59 (50.0)	
2	106 (35.5)	63 (34.8)	43 (36.4)	
3	28 (9.4)	24 (13.3)	4 (3.4)	
4	34 (11.4)	22 (12.2)	12 (10.2)	
EF(%) (mean (SD))	67.20 (12.10)	68.65 (11.16)	65.04 (13.13)	0.012
IVCD (cm) (mean (SD))	1.50 (0.43)	1.44 (0.43)	1.62 (0.41)	0.003
Comorbidities				
DM (%)	198 (48.1)	99 (40.9)	99 (58.2)	0.001
HTN (%)	332 (80.6)	187 (77.3)	145 (85.3)	0.057
Dyslipid (%)	220 (53.4)	130 (53.7)	90 (52.9)	0.956
CAD (%)	173 (42.0)	87 (36.0)	86 (50.6)	0.004
AMI (%)	12 (2.9)	3 (1.2)	9 (5.3)	0.035
CVA (%)	10 (2.4)	4 (1.7)	6 (3.5)	0.372
PAOD (%)	111 (26.9)	53 (21.9)	58 (34.1)	0.008
HF (%)	91 (22.1)	44 (18.2)	47 (27.6)	0.031
COPD (%)	38 (9.2)	22 (9.1)	16 (9.4)	1
LC = 0 (%)	412 (100.0)	242 (100.0)	170 (100.0)	NA
Malignancy (%)	41 (10.0)	27 (11.2)	14 (8.2)	0.419
Arrhythmia (%)	46 (11.2)	25 (10.3)	21 (12.4)	0.629
Amputation (%)				0.026
0	405 (98.3)	241 (99.6)	164 (96.5)	
1.1	2 (0.5)	1 (0.4)	1 (0.6)	
1.2	5 (1.2)	0 (0.0)	5 (2.9)	
Total protein (gm/dL) (mean (SD))	6.78 (0.53)	6.80 (0.51)	6.76 (0.57)	0.45
Albumin (gm/dL) (mean (SD))	3.86 (0.38)	3.92 (0.33)	3.78 (0.42)	<0.001
A.S.T. GOT (IU/L) (mean (SD))	16.44 (10.05)	15.85 (6.93)	17.29 (13.26)	0.153
A.L.T. GPT (IU/L) (mean (SD))	12.67 (11.68)	12.26 (7.17)	13.24 (16.07)	0.407
Alkaline-P (IU/L) (mean (SD))	76.59 (40.74)	73.48 (39.83)	81.03 (41.72)	0.064
Total Bilirubin (mg/dL) (mean (SD))	0.54 (0.22)	0.53 (0.22)	0.55 (0.22)	0.449
Cholesterol (mg/dL) (mean (SD))	156.28 (37.50)	159.12 (35.11)	152.23 (40.43)	0.066
Triglyceride (mg/dL) (mean (SD))	142.82 (110.09)	150.46 (119.93)	131.94 (93.60)	0.093
GlucoseAC (mg/dL) (mean (SD))	115.47 (54.92)	112.51 (52.44)	119.69 (58.18)	0.192
WBC (×1000/μL) (mean (SD))	6.91 (1.94)	6.92 (2.04)	6.91 (1.81)	0.947
RBC (×10^6^/μL) (mean (SD))	3.33 (0.54)	3.36 (0.55)	3.28 (0.52)	0.123
Hb (g/dL) (mean (SD))	10.38 (1.44)	10.50 (1.44)	10.19 (1.42)	0.029
Hct (%) (mean (SD))	31.11 (4.30)	31.43 (4.32)	30.65 (4.24)	0.072
MCV (fl) (mean (SD))	94.06 (7.59)	94.11 (7.80)	94.00 (7.32)	0.881
Platelet (×1000/μL) (mean (SD))	192.60 (58.63)	195.76 (55.65)	188.10 (62.52)	0.192
Fe (μg/dL) (mean (SD))	74.89 (33.08)	76.93 (32.43)	71.99 (33.87)	0.136
TIBC (μg/dL) (mean (SD))	241.33 (48.68)	242.40 (46.26)	239.79 (52.02)	0.593
Ferritin (ng/mL) (mean (SD))	537.89 (277.02)	532.43 (248.82)	545.66 (313.44)	0.634
Tranferrin saturation (%) (mean (SD))	31.35 (12.91)	32.20 (13.25)	30.14 (12.36)	0.112
Al (ng/mL) (mean (SD))	6.86 (3.96)	6.84 (3.94)	6.89 (3.99)	0.891
Post-Dialysis Weight (kg) (mean (SD))	60.21 (13.27)	60.47 (13.43)	59.82 (13.07)	0.625
Uric acid (mg/dL) (mean (SD))	6.32 (1.60)	6.34 (1.64)	6.29 (1.54)	0.725
Na (meq/L) (mean (SD))	138.01 (3.10)	138.20 (2.95)	137.74 (3.30)	0.132
K (meq/L) (mean (SD))	4.63 (0.68)	4.69 (0.64)	4.56 (0.72)	0.066
iCa (mg/dL) (mean (SD))	4.56 (0.51)	4.55 (0.51)	4.58 (0.52)	0.56
P (mg/dL) (mean (SD))	5.13 (1.35)	5.02 (1.25)	5.28 (1.47)	0.051
Kt/V (Gotch) (mean (SD))	1.38 (0.19)	1.39 (0.20)	1.35 (0.19)	0.05
PTH (pg/mL) (mean (SD))	311.25 (313.97)	289.04 (273.17)	342.86 (362.78)	0.087
Ca × P (mg^2^/dL^2^) (mean (SD))	46.88 (13.21)	45.81 (12.66)	48.45 (13.86)	0.048
CXR_AoAC (%)				<0.001
0	120 (31.4)	90 (40.5)	30 (18.8)	
1	83 (21.7)	49 (22.1)	34 (21.2)	
2	107 (28.0)	49 (22.1)	58 (36.2)	
3	72 (18.8)	34 (15.3)	38 (23.8)	
Cardiac/thoracic ratio (%) (mean (SD))	0.52 (0.07)	0.51 (0.06)	0.53 (0.07)	<0.001
Medication				
DDP-4 inhibitor (%)	104 (25.2)	54 (22.3)	50 (29.4)	0.129
TZD (%)	16 (3.9)	7 (2.9)	9 (5.3)	0.326
SU (%)	46 (11.2)	25 (10.3)	21 (12.4)	0.629
MEGLITINIDES (%)	39 (9.5)	19 (7.9)	20 (11.8)	0.244
Alpha-glucose inhibitor (%)	11 (2.7)	1 (0.4)	10 (5.9)	0.002
Insulin (%)	85 (20.6)	33 (13.6)	52 (30.6)	<0.001
GLP-1 (%)	1 (0.2)	0 (0.0)	1 (0.6)	0.859
Statin (%)	137 (33.3)	73 (30.2)	64 (37.6)	0.139
Fibrate (%)	11 (2.7)	5 (2.1)	6 (3.5)	0.551
CCB (%)	232 (56.3)	131 (54.1)	101 (59.4)	0.336
RI/ACEI/ARB (%)	217 (52.7)	117 (48.3)	100 (58.8)	0.046
B-blocker (%)	211 (51.2)	118 (48.8)	93 (54.7)	0.276
Vasodilator (%)	94 (22.8)	55 (22.7)	39 (22.9)	1
Alpha-blocker (%)	46 (11.2)	29 (12.0)	17 (10.0)	0.638
Antiplatelet (%)	199 (48.3)	82 (33.9)	117 (68.8)	<0.001
Anti-coagulants (%)	20 (4.9)	8 (3.3)	12 (7.1)	0.13
Phosphate binders (calcium) (%)	242 (58.7)	149 (61.6)	93 (54.7)	0.196
Calcitriol (%)	173 (42.0)	104 (43.0)	69 (40.6)	0.703
Phosphate binder (non-calcium) (%)	93 (22.6)	50 (20.7)	43 (25.3)	0.323
Interdyalytic hypotension (%)	315 (76.5)	186 (76.9)	129 (75.9)	0.911
No of hypotension episodes (mean (SD))	5.41 (3.26)	5.46 (3.21)	5.34 (3.33)	0.706

## Appendix C

**Table A3:** XGBoost model hyperparameters.

Hyperparameters	Setting
colsample_bytree	0.8
subsample	0.8
booster	gbtree
max_depth	10
eta	0.1
eval_metric	auc
eval_metric	error
objective	binary: logistic
gamma	0.01
lambda	2
min_child_weight	1

**Table A4:** CatBoost model hyperparameters.

Feature	Racy
iterations	1000
thread_count	10
border_count	32
depth	8
eval_metric	AUC
loss_function	Logloss
objective	binary:logistic
logging_level	Silent
l2_leaf_reg	2
random_seed	123
learning_rate	0.001

**Table A5:** LightGBM model hyperparameters.

Feature	Racy
num_leaves	3
thread_count	10
nthread	1
metric	auc
metric	binary_error
objective	binary
min_data	1
learning_rate	0.1
